# What Produces Caries?

**Published:** 1893-06

**Authors:** 


ARTICLE VI.

### WHAT PRODUCES CARIES?

BY DR. SUDDUTH.

The fermenting of meat will not produce decay. Putrefaction will not produce decay. Putrefactive changes of meat will not produce decay. You can only have decay of the teeth, as we understand the term "decay," by the action of one acid, and that is lactic acid. Now I have given a great deal of attention to the food habits of different races in regard to this particular point, because it is an interesting one to study; the food habits of races in connection with the prevalence of decay of teeth, noting how the change of diet will affect the quality of the teeth. Now this change in the quality of the teeth depends upon three things: In the first place, it is a matter of heredity. What I mean is inherited disease. You take the prevalence of syphilis in any one race of people, where it has been first introduced into a race or into a country, and you follow down the history of the teeth of those people, and you will find that the children and grandchildren inherit a lack of lime salts in the bones, that is also reflected in the teeth. The teeth of individuals, whose parents or grand-parents have suffered from syphilis, are very poor in quality. In studying the effect of syphilis on different people, we have that one thing, the deteriorated condition of the general health reflected in the development of the teeth. There is a lack of good development. There is a lack of assimilation on the part of the individual, and it is shown in the teeth just as well as it is in the bones. It is this condition, called rachitis, which is so common.

A change in food habits affects the teeth. You take all carnivorous animals, those that eat meat almost entirely, and decay is almost unknown. Take a dog, for instance, as a typical example of the carnivorous animal. The character of food that a dog eats is self-cleansing. The teeth are clean and white. This food, if it remains in the mouth, and ferments, as I say, will not produce decay. But take a petted dog, poodles and other dogs, fed on dainties and bonbons, and you will find pyorrhea alveolaris and decay. If that is so in regard to the teeth of animals, it is doubly so in regard to the teeth of men. To those people that live on a purely meat diet, like the North American Indians in early days and the Esquimaux down to the present time, decay was almost unknown. You may examine the skull of the primitive races of the Indians of North America, and decay is almost unknown. As they come under the same environment as Europeans and Americans, decay becomes more and more apparent. Change of diet, then, is one of the things that tend to produce decay.

The result of eating starchy food is that it is converted by fermentation in glucose; and further fermentation produces lactic acid before it is taken into the mouth. Now, we know that acid foods, those foods that are acid when taken into the mouth, never produce decay. They produce erosion of the teeth. Take the grape cure that has been used so extensively in Europe, with its fruit acid, and the use of mineral acids; they do not produce decay, they produce erosion. They roughen the surfaces of the teeth, which are more easily abraded in the process of mastication, so that the teeth wear down more quickly. But unless there is eaten the starch, or the glucose itself, through which fermentation is going on in the mouth, decay is not produced. The principal portion of the diet of the Hawaiians, which is poi, glucose would exist before before it is eaten. If they were eating the product as we do, flour and things of that kind, they would have starch taken in the mouth, and the action of the saliva would convert it into glucose, which would then be converted into grape sugar and into lactic acid. The history of all people is, that when they come within the range of civilization, and are dependent on a starch food, decay is more prevalent than it was before. It is common in the East with the servant girls who come from Europe, that after they have been in this country six months or so their teeth begin to decay. It has nothing to do with the change of condition other than as it is affected by the change of diet. A large portion of those servant girls that come from Sweden, Norway, Germany and Ireland come from the farms. They go into the kitchens of our American homes, and there their diet is changed from the self-cleansing, coarse food, which is not starchy and sticky like the food that is prepared in American homes. The results are seen very quickly. Decay sets in and the teeth are broken down. If they developed the same habits of cleanliness that American children are brought up with, and kept their teeth clean, they would not have as much decay as they have; but a tooth-brush among the peasants of Europe is an unknown quantity, and they lose their teeth in this country before they learn what a tooth-brush is for.

Fermentation is constant in the mouth whenever starch is present. We want to distinguish between erosion and decay. Erosion may be caused by general acidity of the saliva, but decay and death, as we understand the term, is the result of fermentation.—*Items of Interest.*

